# Supplementary figures and images for: The alphaherpesvirus conserved pUS10 is important for natural infection and its expression is regulated by the conserved Herpesviridae protein kinase (CHPK)
Source: PLoS Pathog. 2023 Feb 7;19(2):e1010959. doi: 10.1371/journal.ppat.1010959 (PMC9946255; doi:10.1371/journal.ppat.1010959)

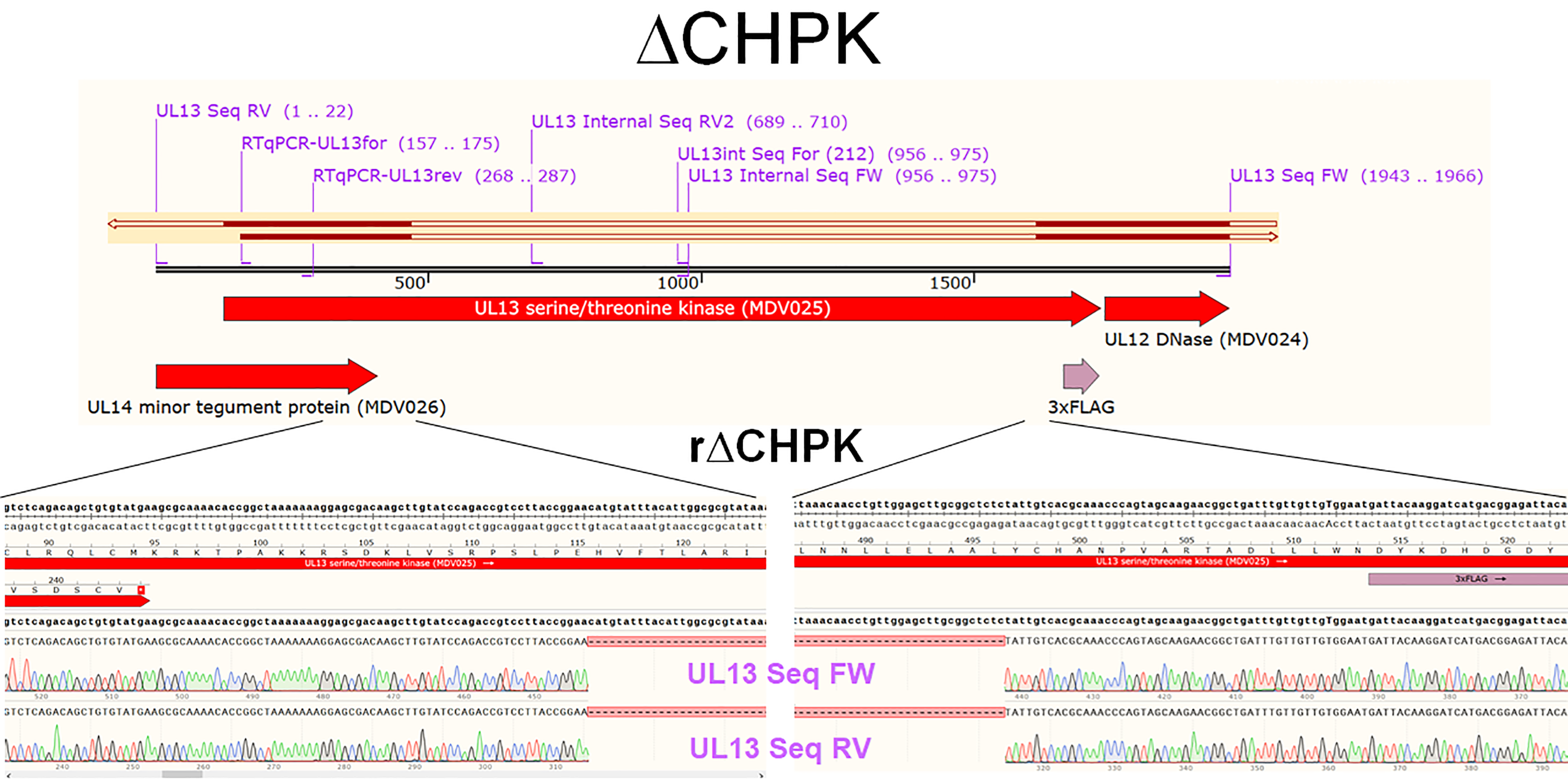

Supplement: S1 Fig — Sanger sequencing was performed on BAC clones using previously published primers [40]. SnapGene software was used for sequencing analysis using its alignment tool. All rΔCHPK BAC clones had the same sequence. Only shown is results for rΔCHPK. (TIF) [file ppat.1010959.s001.tif]

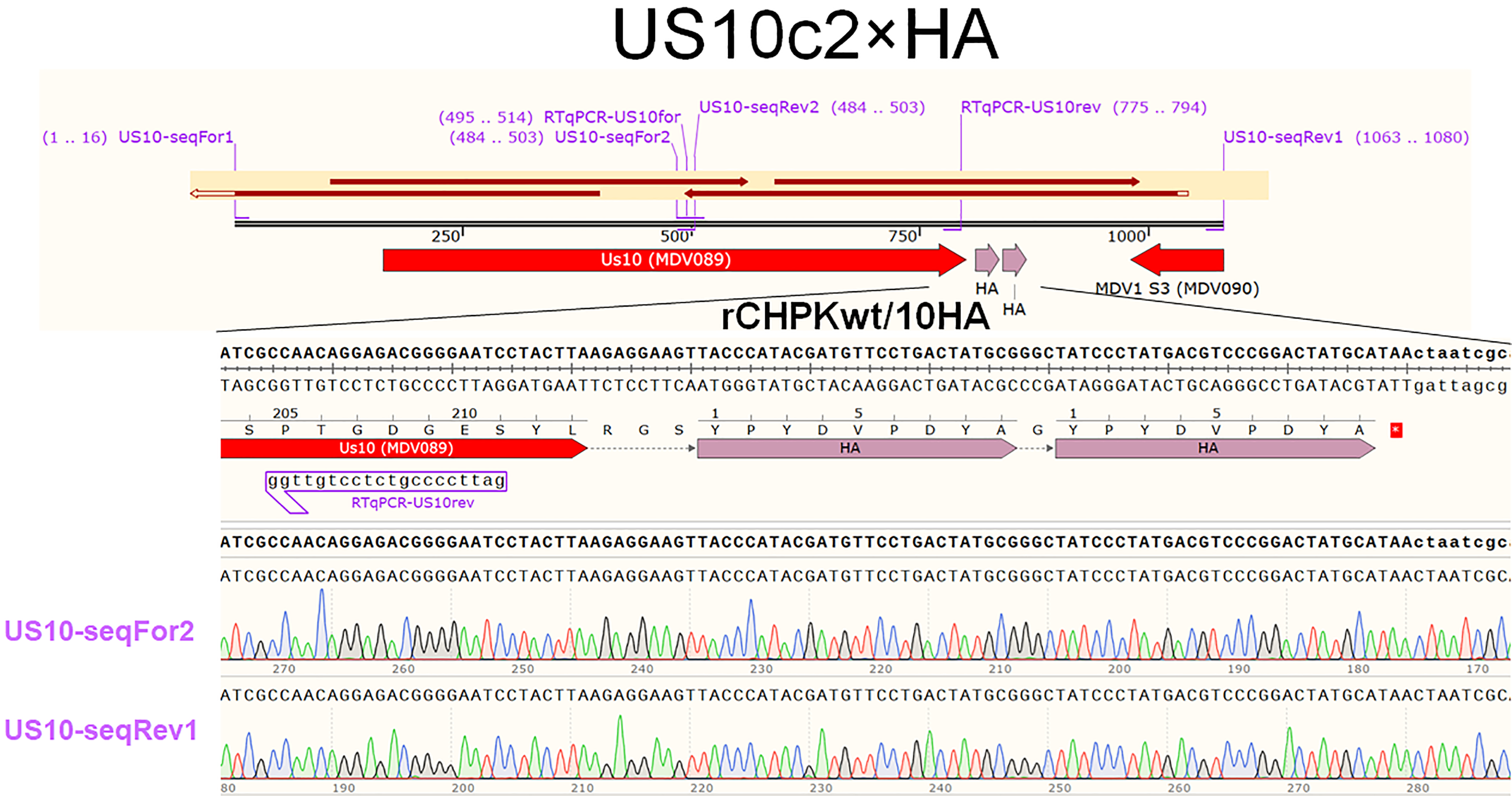

Supplement: S2 Fig — Sanger sequencing was performed on BAC clones using primers shown in S2 Table. SnapGene software was used for sequencing analysis using its alignment tool. Only shown is rCHPKwt/10HA but all other clones had identical sequencing results. (TIF) [file ppat.1010959.s002.tif]

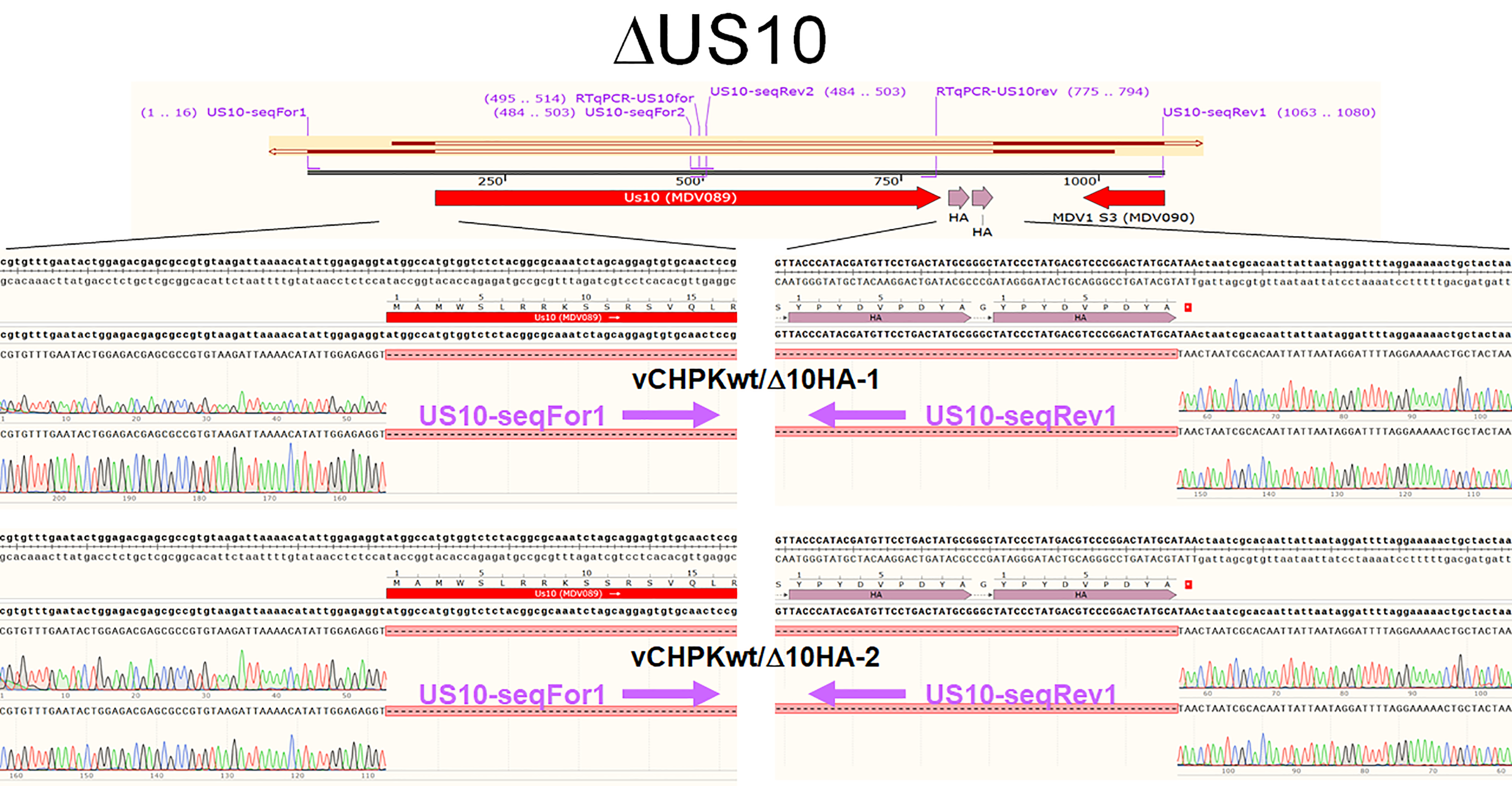

Supplement: S3 Fig — Sanger sequencing was performed on BAC clones using primers shown in S2 Table. SnapGene software was used for sequencing analysis using its alignment tool. Both clones vCHPKwt/D10HA-1 and vCHPKwt/D10HA-2 are shown. (TIF) [file ppat.1010959.s003.tif]

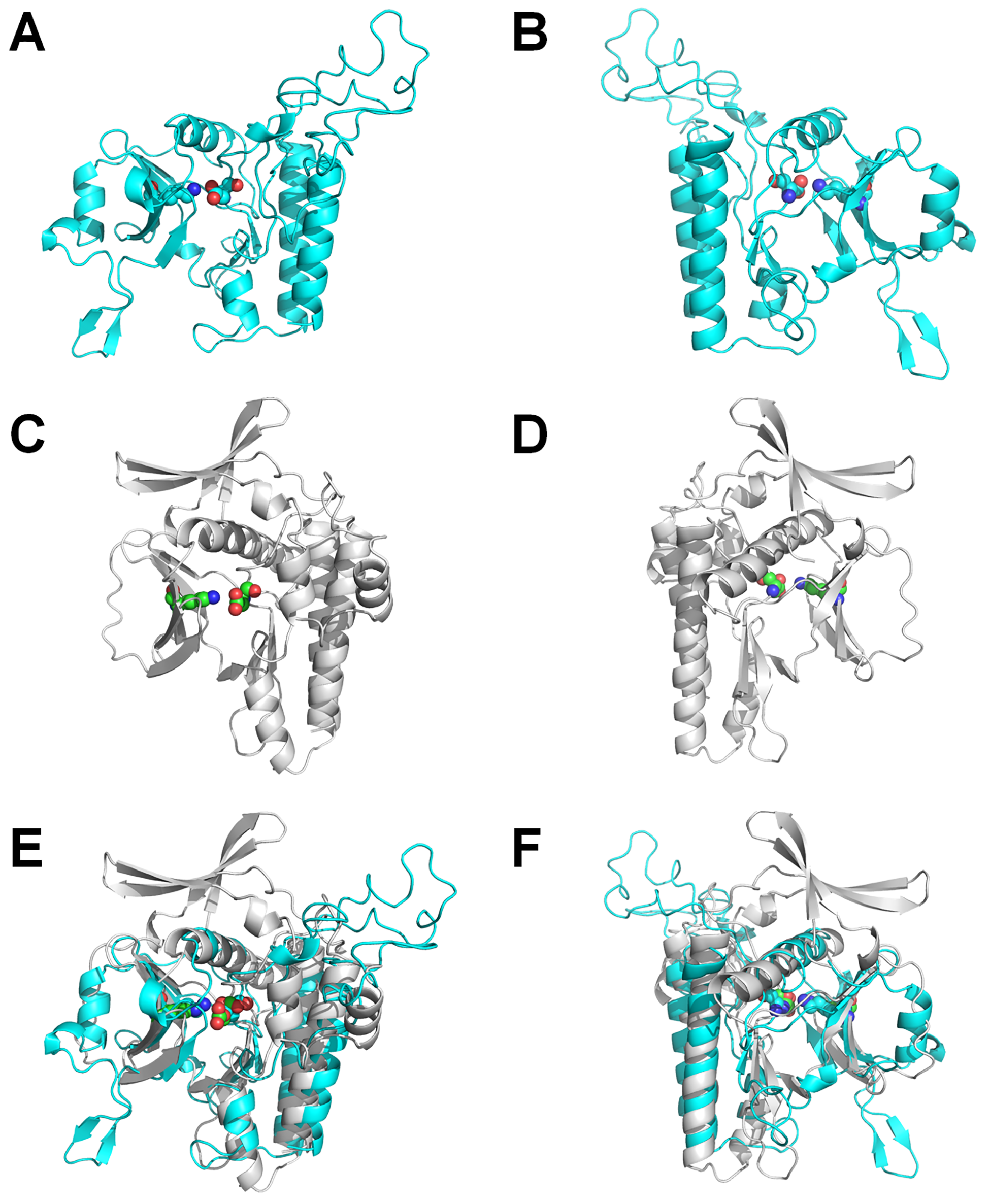

Supplement: S4 Fig — (A-D) The predicted 3D structure from Swiss Model and Alpha fold shown in cyan and grey colors, respectively, with cationic residues of interest LYS170 and ASP293 shown in spheres. (E and F) shows the overlap of both the predicted structures with regions of interest overlapping with 4 Å RMSD. With no major change in the interacting region core structure. Note: Where B, D and F are rotated 180° with respect to A, C, E for better visualization of the protein region of interest. (TIF) [file ppat.1010959.s004.tif]

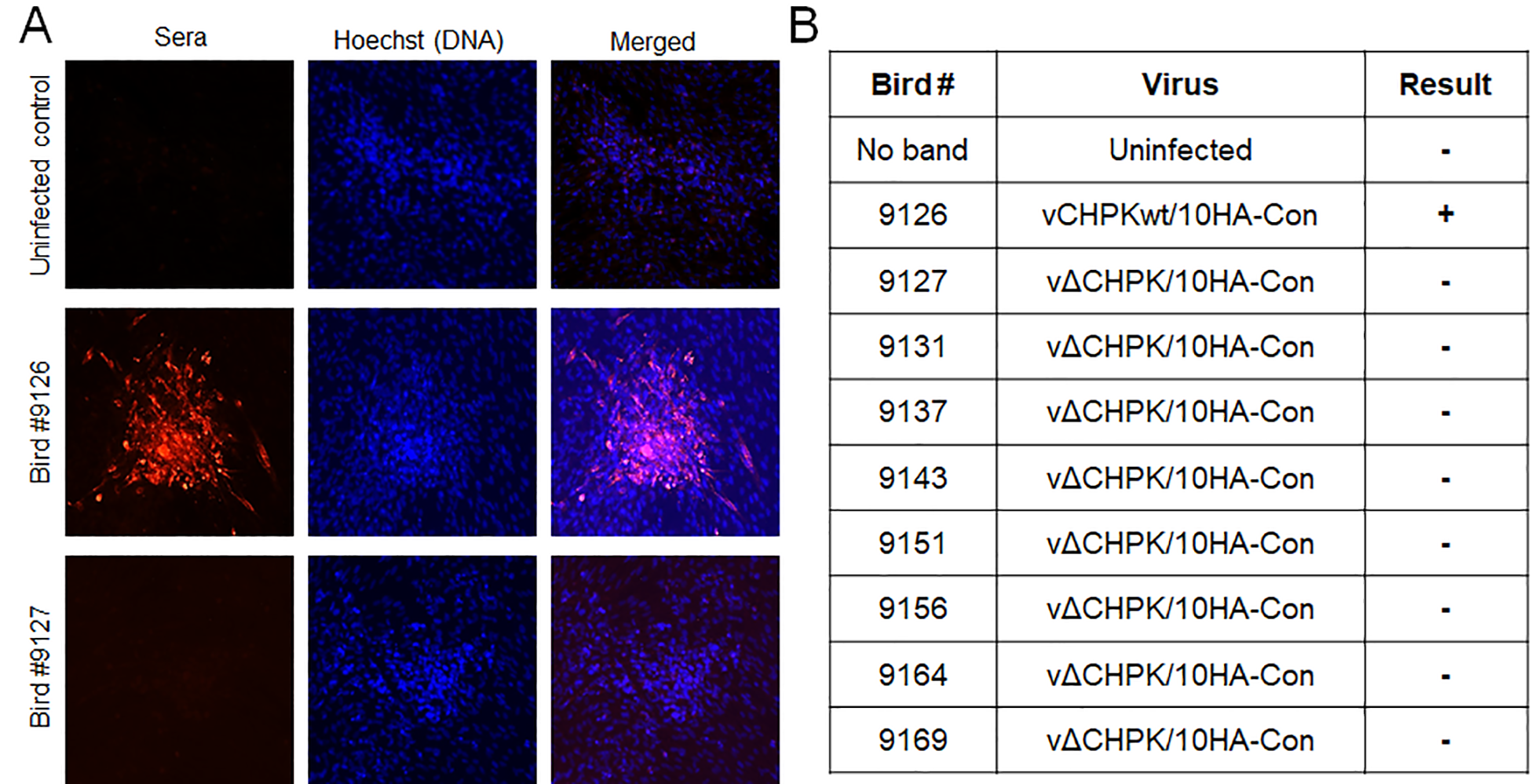

Supplement: S5 Fig — Sera was tested for anti-MDV antibodies using indirect IFAs. (A) Representative images showing the results for uninfected, bird #9126 (vCHPKwt/10HA-Con), and bird #9127 (vΔCHPK/10HA-Con) as negative (-), positive (+), and negative (-) for anti-MDV antibodies. (B) The results of sera collected from uninfected or contact chickens housed with vCHPKwt/10HA or vΔCHPK/10HA-infected chickens in Fig 6B are shown. (TIF) [file ppat.1010959.s005.tif]
