# Supplementary material for: The alphaherpesvirus conserved pUS10 is important for natural infection and its expression is regulated by the conserved Herpesviridae protein kinase (CHPK)
Source: PLoS Pathog. 2023 Feb 7;19(2):e1010959. doi: 10.1371/journal.ppat.1010959 (PMC9946255; doi:10.1371/journal.ppat.1010959)
Supplement: S1 Table — (rMDV). (DOCX) [file ppat.1010959.s006.docx]

**S1 Table. Primers used for generation of recombinant Marek’s disease viruses (rMDV).**

| **Modification*^a^*** |  | **Direction** |  | **Sequence (5’**- **3’)*^b^*** |
| --- | --- | --- | --- | --- |
| ΔUL13  (116-496) |  | Forward |  | gctaaaaaaaggagcgacaagcttgtatccagaccgtccttaccggaatattgtcacgcaaacccagta*tagggataacagggtaatcgattt* |
|  |  | Reverse |  | attccAcaacaacaaatcagccgttcttgctactgggtttgcgtgacaatattccggtaaggacggtct*gccagtgttacaaccaattaacc* |
|  |  |  |  |  |
| US10c2×HA*^c^* |  | Forward1 |  | AGAGGAAGTTACCCATACGATGTTCCTGACTATGCGGGCTATCCCTATGACGTCCCGGACTATGCA*tagggataacagggtaatcgattt* |
|  |  | Reverse1 |  | ATAGCCCGCATAGTCAGGAACATCGTATGGGTAACTTCCTCTTAAGTAGGATTCCCCGTCTC*gccagtgttacaaccaattaacc* |
|  |  | Forward2 |  | TCGGGAATCGCCAACAGGAGACGGGGAATCCTACTTAAGAGGAAGTTACCCATACGATGTTCCTGACT |
|  |  | Reverse2 |  | gtttttcctaaaatcctattaataattgtgcgattagTTATGCATAGTCCGGGACGTCATAGGGATAGCCCGCATAGTCAGGAACA |
|  |  |  |  |  |
| ΔUS10 |  | Forward |  | ttgaatactggagacgagcgccgtgtaagattaaaacatattggagaggtTAActaatcgcacaattatt*tagggataacagggtaatcgattt* |
|  |  | Reverse |  | gttagtagcagtttttcctaaaatcctattaataattgtgcgattagTTAacctctccaatatgttttaa*gccagtgttacaaccaattaacc* |

*^a^*Modification produced in the recombinant virus including deletion of amino acids 116-496 of UL13 (rΔCHPK), addition of 2×HA epitope tag to the C-terminus of US10 (10HA), and removal of US10 (ΔUS10).

*^b^*Red indicates unique upstream integration sequences. Green indicates unique downstream integration sequences. Blue indicates complementary sequences used during resolution of integrates. *Italics indicate the template-binding region of the primers for PCR amplification with pEP-KanS2.*

*^c^*Two PCRs were used to generate the PCR product for integration of the 2×HA at the C-terminus of US10. The first set was used to amplify the I-*SceI-aphAI* cassette from pEP-KanS2 and the second extended the PCR product to include the unique upstream and downstream integration sites using a nest PCR technique.
