# Supplementary material for: The alphaherpesvirus conserved pUS10 is important for natural infection and its expression is regulated by the conserved Herpesviridae protein kinase (CHPK)
Source: PLoS Pathog. 2023 Feb 7;19(2):e1010959. doi: 10.1371/journal.ppat.1010959 (PMC9946255; doi:10.1371/journal.ppat.1010959)
Supplement: S2 Table — (DOCX) [file ppat.1010959.s007.docx]

**S2 Table. Primers used for sequencing, diagnostics, or RT-qPCR assays.**

| **Gene ID** |  | **Locus Tag** |  | **Gene** |  | **Primer Name*^a^*** |  | **Direction** |  | **Sequence (5’**- **3’)** |
| --- | --- | --- | --- | --- | --- | --- | --- | --- | --- | --- |
| 4811486 |  | MDV025 |  | UL13 |  | RTqPCR_UL13for |  | Forward |  | CCAACGGTGGCGAAAACAG |
|  |  |  |  |  |  | RTqPCR_UL13rev |  | Reverse |  | TCGTGAGTTGACGGTTGTCC |
|  |  |  |  |  |  |  |  |  |  |  |
| 4811543 |  | MDV088 |  | US1 |  | RTqPCR_US1for |  | Forward |  | CCGATGTCGCTCGGTTATGT |
|  |  |  |  |  |  | RTqPCR_US1rev |  | Reverse |  | ATGTAGACTTTGGCGTGGGG |
|  |  |  |  |  |  |  |  |  |  |  |
| 4811465 |  | MDV089 |  | US10 |  | US10seqFor |  | Forward |  | acaaacgctgaccccc |
|  |  |  |  |  |  | US10seqIntFor |  | Forward |  | CGGTGTATTGAACGTGCTCC |
|  |  |  |  |  |  | US10seqRev |  | Reverse |  | ccatttacgtccgcctcg |
|  |  |  |  |  |  | US10seqIntRev |  | Reverse |  | GGAGCACGTTCAATACACCG |
|  |  |  |  |  |  | RTqPCR_US10for |  | Forward |  | acgtgctcctcttacacacg |
|  |  |  |  |  |  | RTqPCR_US10rev |  | Reverse |  | gattccccgtctcctgttgg |
|  |  |  |  |  |  |  |  |  |  |  |
| 4811451 |  | MDV092 |  | US3 |  | RTqPCR_US3for |  | Forward |  | acacttcaagcagtacgcga |
|  |  |  |  |  |  | RTqPCR_US3rev |  | Reverse |  | ATGCGTCAGCGGGTTCTTTA |

*^a^*Name of the primers.
