# Supplementary material for: The alphaherpesvirus conserved pUS10 is important for natural infection and its expression is regulated by the conserved Herpesviridae protein kinase (CHPK)
Source: PLoS Pathog. 2023 Feb 7;19(2):e1010959. doi: 10.1371/journal.ppat.1010959 (PMC9946255; doi:10.1371/journal.ppat.1010959)
Supplement: S3 Table — (DOCX) [file ppat.1010959.s008.docx]

**S3 Table. Primers used for generation of expression plasmids.**

| **Construct*^a^*** |  | **Primer Name^b^** |  | **Sequence (5’**- **3’)** |
| --- | --- | --- | --- | --- |
| pcCHPKwt, pcCHPKmut, pcΔCHPK |  | GA_UL13GibsonFragFor |  | tggatatctgcagaattaattccaccacactggactagtgctcatctttgcaagatttggagagtgg |
|  |  | GA_UL13GibsonFragRev |  | ggagacccaagctggctagttaagcttggtaccgagctcgcaaaatggatactgaatcaaaaaacaaaaaaacgacca |
|  |  |  |  |  |
| pcUS10eGFP |  | GA_US10eGFPvectorFor |  | gagacggggaatcctacttaaagggcaattctgcagatatccag |
|  |  | GA_US10eGFPvectorRev |  | cgtagagaccacatggccatggcggcaagggc |
|  |  | GA_US10eGFPfragmentFor |  | gtggaattgcccttgccgccatggccatgtggtctctacg |
|  |  | GA_US10eGFPfragmentRev |  | atatctgcagaattgccctttaagtaggattccccgtctcctgt |

*^a^*Expression construct generated.

*^b^*Name of the primers.
