# Supplementary material for: The alphaherpesvirus conserved pUS10 is important for natural infection and its expression is regulated by the conserved Herpesviridae protein kinase (CHPK)
Source: PLoS Pathog. 2023 Feb 7;19(2):e1010959. doi: 10.1371/journal.ppat.1010959 (PMC9946255; doi:10.1371/journal.ppat.1010959)
Supplement: Attachment — Submitted filename: UL13onUS10Rebuttal(6-I-23).pdf [file ppat.1010959.s009.pdf]

PPATHOGENS-S-22-02306_Response to Reviewers Comments in **Bold**

The alphaherpesvirus conserved pUS10 is important for natural infection and its expression is regulated by the conserved *Herpesviridae* protein kinase (CHPK)

The reviewers considered your manuscript as interesting and well-written, but raised several issues that would need to be addressed in any resubmission. While all reviewer comments would need to be addressed, particularly important ones include additional controls for IP, co-localization, and phosphorylation experiments, excluding secondary mutations as a source of the phenotype of mutant viruses, exploring autophagy as a possibility for the decrease in Us10 protein levels, and eliminating the use of "data not shown".

Reviewer's Responses to Questions

Part I - Summary
Please use this section to discuss strengths/weaknesses of study, novelty/significance, general execution and scholarship.
Reviewer #1: Ponnuraj et al., pursue the very interesting observation that the MDV conserved herpesvirus protein kinase (CHPK) is necessary for natural transmission of the virus and explore the possibility that this function is mediated by phosphorylation of one the virus tegument proteins, pUS10. They present proteomic evidence that pUS10 is phosphorylated at several specific sites in the presence of CHPK catalytic activity, but not in its absence and go on to explore the significance of CHPK and pUS10 in production of infectious virus, virus transmission and development of disease in experimentally and naturally infected chickens.

There are many interesting findings, including that the MDV CHPK catalytic activity is required for improving the specific infectivity of MDV virions, that CHPK strongly affects the expression level of pUS10 at some post-transcriptional level and that CHPK and pUS10 strongly affect each other's localization in the infected cell. The authors also provide suggestive, but not conclusive evidence (see specific comments below), that these two proteins interact physically. The principal finding regarding the functional consequence of pUS10 phosphorylation is negative – deletion of the US10 gene has no significant effect on natural transmission but may possibly promote development of Marek's disease following natural transmission.

1. The co-localization of pUS10 and CHPK looks very convincing in the pair of cells shown in Figure 3E, but it is not clear from the text or legend how many cells were sampled for the quantitative analysis of colocalization. This number should be at least 10 and information should be provided about how cells were selected for analysis to minimize investigator bias. This same comment applies to the colocalization results shown in Figure 5 and 6.

Reply: Thank you for this comment. We do describe the quantitative colocalization procedure in the MM on lines 677-681 of the original manuscript (MS). "Briefly, five randomly selected cells that showed roughly equal expression levels of pCHPK derivatives and pUS10 were chosen, and five regions of interest (ROI) for each image were chosen that had clear concentrations of pUS10. A straight line was drawn that included pUS10 and a profile of pixel intensity for DNA, pUS10, and pCHPK was obtained." However, we did make a mistake in that only three images (with 5 ROIs for each image) were used, therefore; we have corrected this in the text. In all, a total of 15 ROIs per

group were used for analysis. Also, since we are selecting cells with roughly equal expression levels" we removed "randomly selected" on line 677 of the original MS, now line 712 of the revised MS.

Since it was not clear originally, we have included the statement, "…obtained with five regions of interests (ROIs) from three individual cells showing roughly equal expression of pCHPK and pUS10. A represented cell and ROIs are shown in Figure 3E.." in the Results (lines 200-201 of revised MS).

2. The Co-IP shown in Figure 3F is not well-controlled. Showing that VP5 is not co-precipitated goes some way toward demonstrating specificity, but important negative controls are not shown. Ideally, the results of co-IPs in which pUS10 and CHPK are not tagged should be shown.

Reply: Thank you for this comment. We struggle with finding all the optimal controls for these experiments since we lack antibodies specific to most MDV proteins, including pCHPK and pUS10. Thus, performing direct Co-IP of CHPK and US10 without tags is not possible currently. Formerly, we and other groups, have attempted to develop antibodies against MDV CHPK, but have failed in providing the specificity and level of detection needed for such studies. In our stock of antibodies against MDV, the only antibody that works in immunoblots that we would "predict" to not interact with CHPK was VP5. Therefore, we are unable to perform the suggested experiment, but feel this data, in combination with the quantitative co-localization data (Figs 3E, 4CD, and 6F), strongly supports the conclusion that both proteins interact. No changes have been made to the MS.

3. The evidence that the K170M mutation changes CHPK expression is not convincing since the diminished expression of the mutant shown in Figure 3D is not at all evident in the no-inhibitor controls shown in Figure 4B. Indeed, in Figure 4B, it looks like expression of the mutant CHPK is enhanced compared to the wild-type. Since there is evidently variability in the relative expression levels, this effect would need to be carefully quantitated to be convincing. In this light, the molecular dynamics simulation of the effect of the K170M mutation in CHPK is a distraction from the rest of the experimental findings in the manuscript and should be removed.

Reply: Thank you for this comment. We agree the differences in expression in this figure is not as evident as we have previously shown. Therefore, we obtained the average expression levels of pCHPK and pUS10 in three independent experiments with and without the proteasome inhibitor MG132. This data has been included in Fig. 5C with statistical analysis as well. The original immunoblots used for quantitative analysis are also included in the "RawImages" file. After quantification, we could confirm pCHPKmut levels were increased with MG132 treatment, while pCHPKwt and pΔCHPK levels were unaffected. We have modified the results section on lines 236-246 of the revised MS to reflect this. In all, this further analysis has significantly strengthened our conclusions showing quantitatively the differences in expression and the effect of proteasome inhibition on the pCHPKmut levels.

4. The title for the legend of Figure 5 states that pCHPK and pUS10 copurify in cells, but the figure contains no copurification results.

Reply: Thank you for noticing this. We neglected to remove this from the title after rearranging the figures. It has been removed.

5. The authors state that the results show that ". . . tagging MDV pUS10 with 2×HA at the C-terminus did not affect the ability of MDV to reach the FFs, induce MD, and horizontally transmit in chickens . . ."

There is no condition in these experiments in which US10 is not tagged. Thus, the authors can conclude that tagging MDV pUS10 does not prevent MDV from reaching the FFs and so on, but the data leave open the possibility that tagging US10 may delay these events.

Reply: Unfortunately, due to the limited number of experimental rooms and the cost of these experiments, we needed to omit this group; however, the accumulation of data over the years shows the reproducibility of experimental and natural MDV infection in chickens is very high, as long as the same strain of virus, chicken line, and relative inoculation titer is used. Our conclusion is based on a number of published data using the RB-1B strain used in our laboratory and the MD-susceptible Pure Columbian chicken line (refs 23, 69, and 71). Even non-transmittable MDV reaches the FFE in this time frame, while highly attenuated MDV can be severely delayed (see ref 69). Thus, for all the viruses used in our study, the number of birds positive for MDV in the FFEs was >75% and for the case of pUS10 with an HA tag (vCHPKwt/10Ha), this virus reached the feather follicles, and even more importantly, transmitted with similar kinetics as previously published work forming our conclusion. We have included the following sentence to the revised MS (Lines 282-283), "The time to reach the feathers was consistent with former studies examining MDV replication and transmission in PC chickens with >75% birds positive for MDV by 21 day pi [23, 69, 71]."

6. The authors would like to conclude that the two pUS10 bands observed in immunoblotting represent differently phosphorylated forms of the protein, since the lower band is absent in cells infected with the CHPKmut virus. This is reasonable, but not conclusive. Loss of the lower band upon phosphatase treatment is needed to demonstrate this conclusively.

Thank you for this comment. We were reluctant to do these experiments since we had limited amount of protein extracts remaining from in vivo tissues. However, we did have enough protein to perform the dephosphorylation with lambda phosphatase experiment on the same samples we showed in Fig. 9F. This has been added to Fig. 9F and we confirmed the phosphorylated form of CHPK is the lower band and it appears to be the case for pUS10 as well. The following was added to the text of the revised MS, Lines 385-387 ("Dephosphorylation of total protein with lambda (λ) phosphatase confirmed the lower band was indeed the phosphorylated form (pCHPK*), with an abundance of pCHPK* as previously shown [23].") and lines 392-393 ("Dephosphorylation of total protein confirmed the lower band is the phosphorylated form (pUS10*).").

7. The first and second paragraphs of the discussion repeat almost the same information.

Reply: Thank you for noticing this. The first few sentence of the second paragraph (lines 399-403 of original MS) was removed and the beginning of the paragraph re-written (lines 413-416 of the revised MS).

8. The discussion does not address the large difference in specific infectivity in virions made by CHPK and CHPKmut viruses. It would be nice to know what the authors think about this and whether there might be a role for the tegument protein pUS10 in this function of CHPK.

Reply: Thank you for this comment. We did neglect to mention data presented in Figure 1, focusing primarily on the subsequent work. The reviewer is correct difference in infectivity of vCHPKwt and vCHPKmut in Figure 1 is an important finding that should have been discussed. We have included this in the Discussion to reflect this including

Lines 400-401 of revised MS: “Here, we showed that infection of MDV virions produced in chicken epithelial skin cells were considerably less infectious when lacking functional pCHPK.”

Lines 439 of revised MS: “Our cell-free virion infectivity data would support this hypothesis, but…”

Lines 476-479 of revised MS: “Our infectivity studies of MD virions extracted from infected chickens suggests pCHPK is important for infectivity of cell-free MDV and suggests entry into the naïve host during natural infection is the major defect of MDV lacking functional pCHPK.”

Reviewer #2: In this clearly written article by Ponnuraj et al from the Jarolinski Group, the role of the Mareks disease virus conserved herpesvirus protein kinase (CHPK) and one of its potential targets, the US10 gene, are examined in the context of infected cells and in spread of the virus in chickens. The authors go through a number of experiments to show that the protein kinase has an influence on the amount of US10 protein in cell culture and in host animals. They have previously partially detailed the role of the CHPK previously in a publication using a virus with point mutant inactivated protein kinase (ref 23), showing that it has such a role. Here they make a new mutant deleted for a large part of the protein kinase. The identify US10 as a substrate by phosphoproteomics, and show that its phosphorylation is dependent on the viral protein kinase. It interacts with the protein kinase. Virus lacking the US10 protein shows viability and some attenuation in animal models but is surprisingly not that severe. An interesting observation is that lacking US10 changes the cellular distribution of the protein kinase. While overall the work appears to be well done, controlled and has some very intriguing observations, there are some outstanding and unresolved issues that do not outline with clarity the mechanisms going on.

Reviewer #3: In this manuscript, the authors investigated the targets of the conserved herpesvirius protein kinases (CHPK) of Marek’s disease virus (MDV) using LC-MS/MS. They identified the US10 capsid/tegument-associated phosphoprotein as a target of CHPK in MDV infection in vivo. Expression of US10 was shown to be dependent on the CHPK activity. In addition, they examined the role of US10 in this model of virus induced tumor formation and could demonstrate that the protein is dispensable for transmission but plays an important role in the disease induction in naturally infected chickens. The manuscript is very well written and contains exciting data on these two conserved herpesvirus proteins that will be of interest to herpesvirologists in general and beyond. This natural virus-host model provides intriguing data that could not have been obtained for other herpesviruses. Only the following points should be addressed prior to publication.

Part II – Major Issues: Key Experiments Required for Acceptance
Please use this section to detail the key new experiments or modifications of existing experiments that should be <u>absolutely</u> required to validate study conclusions.

Generally, there should be no more than 3 such required experiments or major modifications for a "Major Revision" recommendation. If more than 3 experiments are necessary to validate the study conclusions, then you are encouraged to recommend "Reject".

Reviewer #1: No-tag control for Co-IP shown in Figure 3F.
No-tag control for US10 arrival at follicles, transmission and disease development.
Phosphatase treatment of pUS10 to show multiple bands are due to phosphorylation.

Reply: Please see Part I response.

Reviewer #2: It is not well resolved why intracellular levels of the US10 protein decline with virus point mutated in the CHPK, and why they are nearly non-existent in the CHPK deleted virus. It is apparently not proteosome mediated degradation (not affected by MG132) or at the transcriptional level, but seems to be by another mechanism. This is speculated about in the discussion, but is rather an unfinished. Does stability measured by a protein pulse chase approach show increased degradation of US10 with the lack of CHPK? It may not be just the proteosome, but rather another means of turnover (autophagy). Is the protein being made? The CHPK could be targetting the translation machinery to affect translational levels. How does expression of other late genes become affected? A wider protein study is needed.

Reply: We appreciate this comment. Our laboratory has attempted to address autophagy during MDV infection for almost a decade now but have had no luck in finding commercial antibodies work that in the chicken system. A recent paper showed induction of autophagy in B cells (Trapp-Fragnet, et al Plos Path 2021) and we obtained the antibody used in those studies. However, we did not find positive results in our system (chicken embryo cells) during infection using immunoblotting nor in CECs treated with rapamycin (50 nm, 6h) then bafilomycin A1 (100 nM, 2h) in IFAs where little staining was observed and there was no apparent change following treatment. We have provided these results (Immunoblot and IFA) in the "RawImages" file. Thus, we are unfortunately unable to address this experimentally. We are planning to address the significant change in protein levels as we proceed in this work but feel fully addressing this in the report is beyond its primary scope of addressing US10 in natural infection. No changes have been made to the MS.

Regarding the importance of US10, which is stated as being important for disease induction in chickens, the lack of US10 also affects the cellular localization of the Wt CHPK in cells. As such there are many potentials for lacking the US10 to be the cause of the modest attenuation of the virus in vivo. Thus the role of the US10 with the CHPK is complex and not resolved. It is not clear if the CHPK directly phosphorylates the Us10 or acts through another (cellular?) kinase… also please provide a bit more info on what US10 is in related viruses. Any suggestion of function? Is there a HSV equivalent? The statement made in line 392 is far too strong given the lack of the resolution of the relationship of CHPK and US10.

Reply: Thank you for this comment. We understand the absolute relationship between pCHPK and pUS10 unresolved at this point. However, we feel we have provided significant information on their strong relationship and feel completely resolving the exact regulation could take years. We have not directly identified pCHPK phosphorylating pUS10 using in vitro assays as those experiments take a considerable amount of time and expertise and are also fraught with complex controls and complications. At this point, we felt the MS was getting to expansive and starting to lose focus on the in vivo importance of pCHPK and pUS10. Further studies are surely warranted to directly address the exact mechanism by which pCHPK and pUS10 regulate each other; however, we feel this is beyond the scope of the current paper. We have shown that pUS10 is targeted in vivo through pCHPK (directly or indirectly) that led to our hypothesis that pUS10 was linked to pCHPK requirement for cell free virus infectivity and transmission (where our statement on line 392 or original is derived). However, direct analysis of pUS10 in transmission did not confirm this hypothesis. Our evidence shows direct

regulation of pCHPK and pUS10 at both the post-transcriptional expression and localization. We feel this data forms a strong foundation to further study their co-operative roles in gene expression that could be extended to other herpesviruses. As far as we are aware, all Alphaherpesviruses encode this gene, some encoding two copies, suggesting a broader role in herpesvirus replication. However, very little is known about its function and we believe this work with spark interest in pUS10 homologs for other herpes virologist virtually nothing is known about its role/function.

We have included additional discussion to address the reviewer's comments about potential US10 functions in other herpesviruses on lines 506-524 of the revised MS.

In figure 1, virions are analyzed. However only antyibody blots are shown. It is critical to show a total protein stain of the viruses so one can judge the purity of the virus preparations and the relative abundances of the capsid proteins to other virus proteins, and the consequences of CHPK mutation on the virion protein profile. A blot of a non-structural protein should be shown, which would not be in the virions. And Why is there no major capsid protein seen in the WT infected cell extracts? This is not explained and does not make sense.

Reply: Thank you for this comment. We performed additional immunoblotting with these samples to include MDV pp38 and cellular β-actin antibodies, that we would surmise should not be part of the MD virion. We updated Figure 1C to show this and include the raw images in the "RawImages" file.

As for the lack, or barely detectable capsid protein in the whole cell extract, unfortunately, this was the result of this immunoblot. We typically see more VP5 in the infected cell extracts. During western blotting for MDV pp38, we repeated the VP5 immunoblot and saw more VP5, consistent with our typical results where we see VP5 abundantly detected in both FFE and virion preps. This result underscores the virion prep immunoblots though, as we show the levels of virion proteins are abundant. Although not highly purified, we are clearly isolating and concentrating virions in the extracts based on both immunoblotting and infectivity assays.

The following sentence was included in the text on lines 140-142 of the revised MS," Immunoblotting for MDV pp38 and cellular β-Actin, both expected to be absent in MD virions, were used as controls for virion extract purity (Fig 1C) and both were absent in the virion preparation."

Reviewer #3: 1) The authors generated an exciting set of recombinant viruses for their study. Unfortunately, they did not exclude the presence of secondary mutations in these mutants. This would be particularly important for the US10 mutant that provided very important data on the role of this protein in a natural infection. The authors (e.g. Dr. Spatz) should sequence at least this crucial mutant to ensure that this exciting phenotype is not due to a secondary mutation that can occur during mutagenesis.

Reply: We and other researchers have previously sequenced numerous engineered avian herpesvirus genomes that were mutated using the highly precise two-step Red recombination technique and never found any significant secondary site changes. An example of this sequencing exercise to confirm the whole genome sequences of altered RB1B BACs can be found in Chuard A et al. J Virol. 2020 Dec 22;95(2). The secondary site changes invariantly occur in homopolymer stretches, a resolution shortcoming of next-generation sequencing technologies. Overall Sanger sequencing of PCR amplicons targeting these stretches affirm that the two-step Red recombination altered genomes possessed no

secondary mutations that would affect the phenotypes. This characterization of final constructs using Sanger sequencing of the target region in lieu of cost-prohibited whole genome sequencing is also exemplified in Previdelli RL et al. Viruses. 2019 Jan 28;11(2):111. We agree that sequencing a "dead" virus would be required to ensure no other genes were affected during the generation of the mutants. However, in our case, we saw little difference in the vCHPKwt/10HA and vCHPKwt/Δ10HA during experimental infection (Fig. 9), suggesting no additional changes were made. Because of this and the negative cost-to-benefit ratio of sequencing whole herpesvirus genomes (Quote of ~$6,000 to sequence 10 BAC clones) we do not feel sequencing BACs are worthwhile for this project. No changes were made to the revised MS.

Part III – Minor Issues: Editorial and Data Presentation Modifications
Please use this section for editorial suggestions as well as relatively minor modifications of existing data that would enhance clarity.

Reviewer #1: Legend for Figure 6A should specify abbreviations. After a minute of confusion, I figured out that the -Con suffix denoted the results for chicken infected by contact with experimentally infected chickens, but this information should be in the legend.

Reply: Thank you for noticing this. We have included the abbreviation in the legend of revised MS.

Reviewer #2: There are many sections that contain statements with data not shown. This does not conform with PLOS policy. Either the data is shown or the statement must be removed.

Reply: We have now included the former "data not shown" as supplemental figures to conform to PLOS policy.

S1-3 Figs show sequencing results for BAC clones and "data not shown" (lines 170-171 of original MS) was replaced with "S1-3 Fig" in the revised MS (lines 173-174). The sequence program SnapGene software was also included in the MM on lines 554-556 of the revised MS and mentioned in the Figure captions.

S4 Fig shows the predicted 3D structures of pCHPKwt and pCHPKmut using both Swiss-Model and Alpha Fold programs.

S5 Fig shows anti-MDV responses in the sera and description of the assays is shown in MM (lines 783-790 of revised MS). "Data not shown" on line 280 of the original MS was changed to "S5 Fig" on line 292 of the revised MS.

A lot of the first part of the work seems to be confirmatory of that published in reference 23, where it was shown that the CHPK influenced the in vivo spread and replication of MDV. This reduces importance and novelty of the findings presented here. By the looks of it, figure 1A largely is repetitive of that seen in publication 23. Please specify clearly which work is not precedented and what repeats previously published work.

Reply: Thank you for this comment. The reason for including this information is three-fold. First, we wanted to confirm our former studies with different samples for reproducibility. Second, we wanted to show data from the same chickens with respect to EM, immunoblotting, and infectivity for a well-controlled experiment. Also, the previous experiments did not include infectivity data that is very important here. Third, since MDV is not an overly popular pathogen, we felt it would be important for the reader to visualize the experiment without having to go back to previous work. We are not clear in this though and have amended the information. We have included the following statement, "To address this, additional samples were collected from chickens infected with (v)CHPKwt or vCHPKmut from our previous experiment, and skin/feather samples were collected to examine the structure of virions within FFE skin cells and their infectivity." on lines 122-125 of the revised MS.

166-170 details the BAC analyses. However it would be helpful to add the restriction sites for the regions concerned to the figure.

Reply: We have included locations of AfeI and HindIII restriction sites in Figure 2A using triangles, with purple as AfeI and HindIII as orange. This has been included in the figure legend as well.

Line 182 please show the single fluor channels as the nuclear location is not obvious in the figure

Reply: We have now included the DNA, pCHPK, and pUS10 fluor channels individually, as well as all three merged for Fig. 3C.

Have the authors considered making a virus that has the US10 protein that simply lacks the sites of phosphorylation (esp the SPKE mutated to alanine, and seen if this virus is of the same phenotypes as the US10 deletion?

Reply: We had originally planned to do those experiments immediately, assuming US10 was required for transmission. It is possible the phosphorylation sites contribute to the decreased disease incidence during natural infection. However, this would require a significant amount of time and money to address and felt this more suited for a separate study. We do mention the lack of these experiments in the Discussion (lines 467-470 of original MS) "…the potential positive effect of phosphorylation on pUS10 functions is diminished; however, the close regulatory relationship between pCHPK and pUS10 provides an alternative hypothesis that pCHPK phosphorylation of pUS10 has an effect on pUS10 levels." No changes have been made to the revised MS.

Figure 4 a needs a normalizing gene expected to be expressed by both viruses. could rt pcr signal for either gene come from a run on transcript from either side? This is very common in the herpesviruses. The RNA seq data not shown (in a different paper?) could help explain the transcription of these genes without the functional CHPK.

Reply: Thank you for this comment. The reviewer makes an excellent point about run on transcripts. In fact, it is believed the US10 ORF is part of a bicistronic transcript of US1 (ICP22) and US10 (Boumart et al, J Gen Virol. 2018). Therefore, we now include US1, along with US3 that lies within the US region like US10, and two additional genes UL47 and UL49 in Figure 5A (formerly 4A). There were no differences between all genes further suggesting the decreased protein level for UL13 (CHPK) and US10 is not due to an overall decrease in transcription in infected cells. Primer information was included in S2 Table and the MM. The following sentence was included in the Results (Lines 226-228 of revised MS), "We also included US1, US3, UL47, and UL49 mRNA levels and found no differences in

viral transcription of these genes as well, suggesting pCHPK plays no role in viral gene transcription."

Figure 5A it is clear to this reader that the magnification of CHPK mutation is different from that of the WT and deletion mutants. Either that, or the mutation has a consequence on nuclei size!!!! And only a single infected cell is shown. Yet the cellular distribution patters seem quite variable in infected cell culture….

Reply: Yes, the scale bar shows the difference in magnification; however, this is not an infected cell, but a transfected cell. No changes have been a made to the MS.

Did the authors look autophpagy as a basis for the loss of ORF10.

Reply: Please see our response above.

There was no other mention of other targets for the CHPK in the phosphoproteome analyses studies……

Reply: These results are in the process of being put together in a large MS and should be out early in 2023. We included the US10 data as it was confirmatory for our preliminary data on US10 that suggested our further analysis of US10. No changes have been made to the MS.

Reviewer #3: 1) Line 177ff & Figure 3: The authors identify that US10 expression severely impaired (Fig. 3B). But instead of continuing with this aspect, they jump to the localization of CHPV and its mutant (Fig. 3C). Only then they come back to the US10 expression validating it with a western blot (Fig. 3D). To keep the story line straight, the authors could optimally show the western blot earlier as 3C before addressing the CHPV localization.

Reply: Thank you for this comment. We agree, it would be better to continue with addressing co-localization then move onto stability. We have re-organized and the order of Figures 4 and 5 are switched. Additional changes throughout the MS reflect this modification.

2) The authors use the Swiss-Model engine to predict the structure of the CHPK as no crystal structure is available. They should reevaluate their prediction with AlphaFold 2 as this has a much greater accuracy with its AI-based predictions.

Reply: We thank the reviewer for the suggestion. We performed the prediction using Alpha Fold and its predicted core 3D region was identical to that of the Swiss Model used in the current work. We have added one sentence in the main text and added a supplementary figure (S4 Fig) on Alpha Fold prediction. The sentence beginning with "As the crystal structure…" on line 223 of the original MS was modified to include this analysis (now line 248 of the revised MS).

3) In their in vivo experiments, they only show the disease incidence, but not the tumor incidence. It would be great if they could provide this data in their manuscript, as they certainly have it. At least they should state if all animals that developed disease also had tumors.

Reply: The reviewer is correct that all chickens that showed clinical signs of MD also had tumors. We have included the statement (lines 288-289 of revised MS) in the results; "All chickens showing clinical signs had gross lesions following necropsy" and labeled the y-axis in Figs. 6B and 9C as "Percent (%) MD (tumor) incidence" to reflect this.

Additional changes:

- **We have moved Tables 1-3 to Supplement information (S1-S3 Tables) to reduce the size of the MS.**
- **Scale bar sizes were noted in the legend for all microscopy images in case the labeling is not adequate.**
- **Two additional references (71 and 80) were added, altering the number of the references from the original.**
- **Small changes made are highlighted in yellow that consisted of having conformity in abbreviations and descriptions (e.g., CECs, pCHPK, Ly6E, Actin, etc.) or for descriptive clarity (e.g., "virtually" on line 191, "protein" on line 223, etc.) or grammar (e.g., "days" on line 361, "with" on line 382, "observed" on line 389, etc.).**
- **Author Contributions were added to the MS.**
- **Citation of BioRender.com was included in the Figure 7 legend.**
